# Diagnostic performance and clinical feasibility of a novel one-step RT-qPCR assay for simultaneous detection of multiple severe acute respiratory syndrome coronaviruses

**DOI:** 10.1007/s00705-022-05383-0

**Published:** 2022-02-09

**Authors:** Tran Bac Le, Hye Kwon Kim, Min-Ju Ahn, Mark Zanin, Van Thi Lo, Shiman Ling, Zhanpeng Jiang, Jung-Ah Kang, Pan Kee Bae, Yeon-Sook Kim, Seungtaek Kim, Sook-San Wong, Dae Gwin Jeong, Sun-Woo Yoon

**Affiliations:** 1 Bio-nanotechnology Research Center, Korea Research Institute of Bioscience and Biotechnology, Daejeon, South Korea; 2 Bio-Analytical Science Division, University of Science and Technology, Daejeon, South Korea; 3 Chungbuk National University, Cheongju, South Korea; 4 State Key Laboratory for Respiratory Diseases, Guangzhou Medical University, Guangzhou, Guangdong Province China; 5 School of Public Health, The University of Hong Kong, Hong Kong, China; 6 BioNano Health Guard Research Center, Korea Research Institute of Bioscience and Biotechnology, Daejeon, South Korea; 7 Chungnam National University School of Medicine, Daejeon, South Korea; 8 Institute Pasteur Korea, Seongnam-si, South Korea

## Abstract

Coronavirus disease 2019 (COVID-19) is an acute respiratory infection caused by severe acute respiratory syndrome coronavirus 2 (SARS-CoV-2). Other coronaviruses (CoVs) can also infect humans, although the majority cause only mild respiratory symptoms. Because early diagnosis of SARS-CoV-2 is critical for preventing further transmission events and improving clinical outcomes, it is important to be able to distinguish SARS-CoV-2 from other SARS-related CoVs in respiratory samples. Therefore, we developed and evaluated a novel reverse transcription quantitative polymerase chain reaction (RT-qPCR) assay targeting the genes encoding the spike (S) and membrane (M) proteins to enable the rapid identification of SARS-CoV-2, including several new circulating variants and other emerging SARS-like CoVs. By analysis of *in vitro*-transcribed mRNA, we established multiplex RT-qPCR assays capable of detecting 5 × 10° copies/reaction. Using RNA extracted from cell culture supernatants, our multiple simultaneous SARS-CoV-2 assays had a limit of detection of 1 × 10° TCID_50_/mL and showed no cross-reaction with human CoVs or other respiratory viruses. We also validated our method using human clinical samples from patients with COVID-19 and healthy individuals, including nasal swab and sputum samples. This novel one-step multiplex RT-qPCR assay can be used to improve the laboratory diagnosis of human-pathogenic CoVs, including SARS-CoV-2, and may be useful for the identification of other SARS-like CoVs of zoonotic origin.

**Supplementary Information:**

The online version contains supplementary material available at 10.1007/s00705-022-05383-0.

## Introduction

Coronaviruses (CoVs) are single-stranded, positive-sense RNA viruses belonging to the family *Coronaviridae* that can infect both animals and humans [1–3]. The subfamily *Orthocoronavirinae* of the family *Coronaviridae* is divided into four distinct genera: *Alphacoronavirus*, *Betacoronavirus*, *Gammavoronarius*, and *Deltacoronavirus.* Alpha- and betacoronaviruses are associated with human infection, and seven CoVs, including severe acute respiratory syndrome (SARS)-CoV, Middle East respiratory syndrome (MERS)-CoV, and SARS-CoV-2, the causative agent of the ongoing pandemic, have been shown to cause respiratory diseases in humans [4, 5]. *Sarbecovirus*, a subgenus of the genus *Betacoronavirus*, includes SARS-CoV, SARS-CoV-2, and other related animal viruses [5].

In December 2019, cases of pneumonia of unknown origin were reported in the city of Wuhan, Hubei, China [5]. A novel betacoronavirus, which was later officially named SARS-CoV-2, was discovered to be the causative agent of coronavirus disease 2019 (COVID-19) [6]. Next-generation sequencing analysis of the whole SARS-CoV-2 genome revealed that the virus shares 80–89% nucleotide sequence identity with SARS-CoV. However, the genome sequence identity between SARS-CoV-2 and bat-derived SARS-like CoVs, such as BetaCoV/bat/Yunnan/ RaTG13/2013, is approximately 96%, suggesting that SARS-CoV-2 may have originated in a bat species. Therefore, pan-SARS-CoV molecular diagnostics are no longer used to detect SARS-CoV-2, and international research groups have been designing specific primers and probes for the detection of SARS-CoV-2 based on the most conserved genes, such as the RNA-dependent RNA polymerase (RdRp), envelope (E), or nucleocapsid (N) gene [7]. Initial RT-qPCR assays were based on detection of a single target gene; however, assays for detecting SARS‐CoV‐2 RNA are now available targeting one, two, and/or three viral genes, resulting in complicated diagnostic criteria for positive and negative results [8–10]. For example, verification of positive detection of SARS‐CoV‐2 requires all target genes to be positive, including the RdRp, N, and E genes [11, 12]. However, primers targeting the RdRp gene for detection of SARS-CoV-2 cross-react with SARS-CoV RNA [11], and some researchers have found that the sensitivity of RT-qPCR is only approximately 70% for SARS-CoV-2 diagnosis [13], which may result in false-negative results. These results suggest that, although a protocol using RT-qPCR assay for SARS-CoV-2 detection using RdRp, E, and N genes has been established, alternative molecular diagnostic targets are needed to improve the sensitivity of SARS-CoV-2 virus detection.

The aim of this study was to develop a novel RT-qPCR assay to rapidly detect SARS-CoV-2 by targeting the genes encoding the spike (S) and membrane (M) proteins, effectively distinguishing SARS-CoV-2 from other bat-related SARS-like CoVs. The rationale behind this approach was not only to design a highly sensitive and specific primer and probe set for the detection of SARS-CoV-2 but to also produce a tool that would be able to distinguish this virus from other similar SARS-like CoVs, such as those found in the bat reservoir, which would have utility for use in resource-poor regions with limited laboratory capacity. The S gene encodes a trimeric protein, with each monomer comprised of an S1 (receptor-binding) and an S2 (membrane-fusion) subunit [14]. Although several amino acid substitutions [15] and deletions [16] have been reported in the S1 subunit of SARS-CoVs, the amino acid sequence of the S2 subunit is conserved, similar to those of other CoV genes, including the RdRp, E, and N genes. Accordingly, the S gene represents a molecular diagnostic target for SARS-CoV-2 detection in nasopharyngeal or throat swabs, as has been demonstrated in previous studies [17, 18]. The M gene is a conserved gene encoding an important glycoprotein that functions to maintain virion size and shape and assists in the assembly of other structural proteins [19]. Moreover, the M gene has been shown to be useful for applications in molecular diagnostics for viral pathogen detection [20, 21]. However, the M gene has not been used in screening procedures for SARS-CoV-2 detection.

In this study, we developed and assessed the sensitivity and specificity of an RT-qPCR assay targeting the S2 subunit of the S gene and the M gene for simultaneous detection of SARS-CoV-2 and SARS-like CoVs and avoidance of false-negative results. Overall, our novel one-step multiplex RT-qPCR assay was found to be sensitive and specific, and it showed utility in the specific detection of human-pathogenic members of the subgenus *Sarbecovirus*, including SARS-CoV and SARS-CoV-2.

## Materials and methods

### Primer and probe design for RT-qPCR

To develop the RT-qPCR assay for detecting various types of CoVs, four specific probes targeting the S (S2 domain) and M genes of SARS-CoV-2 and SARS-like CoVs were developed. To inform the design, we obtained 265 reference sequences from the GenBank and Global Initiative on Sharing All Influenza Data (GISAID) databases, which included 200 reference sequences representing 10 clades of SARS-CoV-2 and six variants of concern worldwide (up to April 5, 2021), 25 reference sequences of SARS-CoV, and 40 reference sequences of nonhuman SARS-related viruses detected in bats and pangolins (isolated between 2005 and 2019). The sequences were aligned using ClustalW in BioEdit software (version 6.0) and used as a template for the design of the multiplex RT-qPCR assay. The probes were labeled with 5-carboxyfluorescein (FAM) and 5-hexachlorofluorescein (HEX) for detection of the M gene from SARS-CoV-2 and SARS-like CoVs, respectively, and with cyanine dye 5 (Cy5) and Texas red for detection of the S gene (S2 domain) of SARS-CoV-2 and SARS-like CoVs, respectively. RT-qPCR targeting the RdRp and E genes was used as a gold standard for evaluating the results obtained with our assays. All primers and probes, including reference primers and probes targeting the E and RdRp genes [22] were synthesized by Cosmogenetech, Inc. (Seoul, Republic of Korea).

### Viruses and viral RNA samples

To evaluate the sensitivity and specificity of our multiplex RT-qPCR assay, cell culture supernatants from cells infected with CoVs and other respiratory viruses were used. In total, 19 different cultured viruses were evaluated. Four SARS-CoV-2 isolates from Korea (wild-type, alpha variant, beta variant, and delta variant), human coronavirus NL63 (Korea/CN0601/14), human coronavirus 229E (Korea/KUMC-9), human coronavirus OC43 (KBPV-VR-8), and MERS-CoV (MERS-CoV/KOR/KNIH/002_05_2015) were obtained from the Korea Centers for Disease Control and Prevention (KCDC, Republic of Korea), and other viruses, including porcine epidemic diarrhea virus (Korea/SM98), dengue virus type 2 (Korea/DENV-2/KBPV-VR-29), human H3N2 influenza A virus (Korea/37/2012), human H1N1 influenza A virus (California/04/09), human influenza B virus (B/Brisbane/60/2008), parainfluenza virus 1 (Korea/KUMC-44), human respiratory syncytial virus (HRSV-A/IC688/12), and adenovirus type 3 (KUMC-62), were obtained from the Korea Research Institute of Bioscience and Biotechnology (KRIBB) or purchased from the Korea Bank for Pathogenic Viruses (Republic of Korea) [23]. RNA from SARS-CoV (HKU-39849) was provided by Dr. Seungtaek Kim (Institute Pasteur Korea) [24], and viral RNA samples from bat-derived SARS-related CoV (GenBank accession numbers: MK991935 and MK991936) from bat fecal samples, which were used in specificity assays (Table 2), were provided by Prof. Hye Kwon Kim (Chungbuk National University, South Korea) [25].

### SARS-CoV-2 propagation

Vero cells were purchased from the American Type Culture Collection (cat. no. CRL-1586; Manassas, VA, USA) and were maintained in Dulbecco’s modified Eagle medium (Corning, NY, USA) containing 5% fetal bovine serum (Thermo Fisher Scientific, MA, USA) and penicillin (100 IU/mL; Thermo Fisher Scientific) with incubation at 37 °C in an atmosphere containing 5% CO_2_. For titration of SARS-CoV-2, Vero cells were seeded at 1 × 10^5^ cells/well in 96-well plates for 24 h before inoculation with serial tenfold dilutions of SARS-CoV-2. After incubation for 72 h, the 50% tissue culture infective dose (TCID_50_)/mL was determined using the method described by Reed and Muench [26]. All SARS-CoV-2 experiments were conducted at KRIBB (Daejeon, Republic of Korea) with approval from and in accordance with the guidelines of the Institutional Biosafety Committee (approval number KRIBB-IBC-20200208). Experimental work with SARS-CoV-2 was conducted in a biosafety level 3 (BL-3) facility at KRIBB (permission number KCDC-HP-19-3-01).

### One-step multiplex RT-qPCR assay

Viral RNA was purified using a QIAamp Viral RNA Mini Kit (QIAGEN, Hilden, Germany), following the manufacturer’s instructions, and samples were stored at -80 °C until further analysis. Viral RNA from SARS-CoV-2 was extracted from 200 μL of culture supernatant in a BL-3 containment facility at KRIBB, and all RT-qPCR assays were conducted in BL-2 facilities at KRIBB. RT-qPCR assays were performed using a LightCycler 96 instrument and analysis software (Roche, Switzerland). We used a SensiFAST Probe No-ROX One-Step Kit (Bioline, UK) for the one-step RT-qPCR assays according to the manufacturer’s guidelines. Briefly, the reaction mixture contained 5 μL RNA template, 0.2 μL of reverse transcriptase, 0.4 μL of RNase inhibitor for cDNA synthesis, 10 μL of 2× reaction buffer with Taq polymerase, 1 μL of each primer (10 µM), 1 μL of each probe (5 μM), and RNase-free water to a final volume of 20 μL. The PCR cycling conditions were as follows: initial incubation at 45 °C for 15 min and 95 °C for 10 min for the reverse transcription step, followed by 40 cycles at 95 °C for 5 s and 58 °C for 30 s.

### Determination of the detection limit based on *in vitro*-transcribed RNA and SARS-CoV-2 virions

To evaluate the detection limit of the one-step multiplex RT-qPCR assay for *in vitro*-transcribed RNA, DNA segments from wild-type SARS-CoV-2 (hCoV-19/South Korea/KUMC01/2020), which covered both the S2 domain and M gene, were amplified by one-step RT-PCR using primers containing the T7 promoter (CoV-2_T7_24525_Forward, 5′-TAATACGACTCACTATAGGGTTGATCACAGGCAGACTTC-3′; and CoV-2_26894_Reverse, 5′-TCTGGTCAGAATAGTGCCATGGA-3′) and a One-Step RT-PCR Kit (QIAGEN, Germany) following the manufacturer’s guidelines. DNA purified using a gel extraction kit (QIAGEN, Germany) was subjected to mRNA transcription using an mMESSAGE mMACHINE T7 Transcription Kit (Thermo Fisher Scientific, USA) following the manufacturer’s guidelines. Briefly, the transcription mixture contained 200 ng of DNA template, 10 µL of 2× NTP/CAP solution, 2 μL of 10× reaction buffer, 2 μL of enzyme mix, and RNase-free water to a final volume of 20 μL. The reaction conditions were as follows: incubation at 37°C for 2 h, addition of 1 μL TURBO DNase to the reaction mix, and incubation for 15 min at 37 °C to remove the DNA template. The purified mRNA preparation had a concentration of 263 ng/µL, which was calculated to be 2 × 10^11^ copies/µL using an online tool (https://endmemo.com/bio/dnacopynum.php). The transcribed mRNA was serially diluted tenfold from 10^7^ to 10^0^ copies/µL and then evaluated by RT-qPCR. To assess the sensitivity of the one-step multiplex RT-qPCR assay for SARS-CoV-2 virions, SARS-CoV-2 was serially diluted tenfold from 10^6^ to 10^0^ TCID_50_/mL, and viral RNA was then extracted using a QIAamp Viral RNA Mini Kit (QIAGEN, Germany) according to the manufacturer’s instructions. The intact viral RNA was then amplified by RT-qPCR.

### Collection of human clinical samples

Virus-negative nasal swabs and sputum samples were obtained from healthy donors, and clinical samples were obtained from patients with COVID-19 at Chungnam National University School of Medicine (Daejeon, South Korea). In total, 67 clinical samples (40 from men and 27 from women) were used in this study, including 29 from patients with laboratory-confirmed COVID-19 who tested positive for SARS-CoV-2 RNA by RdRp2 assays and 38 healthy donors (age: 18–64 years). Written informed consent was obtained from all participants. All samples were collected with the approval of the Institutional Review Board (IRB) of the National Medical Center, Republic of Korea (IRB number: 2020-03-057). All experiments using human clinical samples were performed following approved guidelines.

## Results

### Primer and probe design for RT-qPCR

**Fig. 1:**
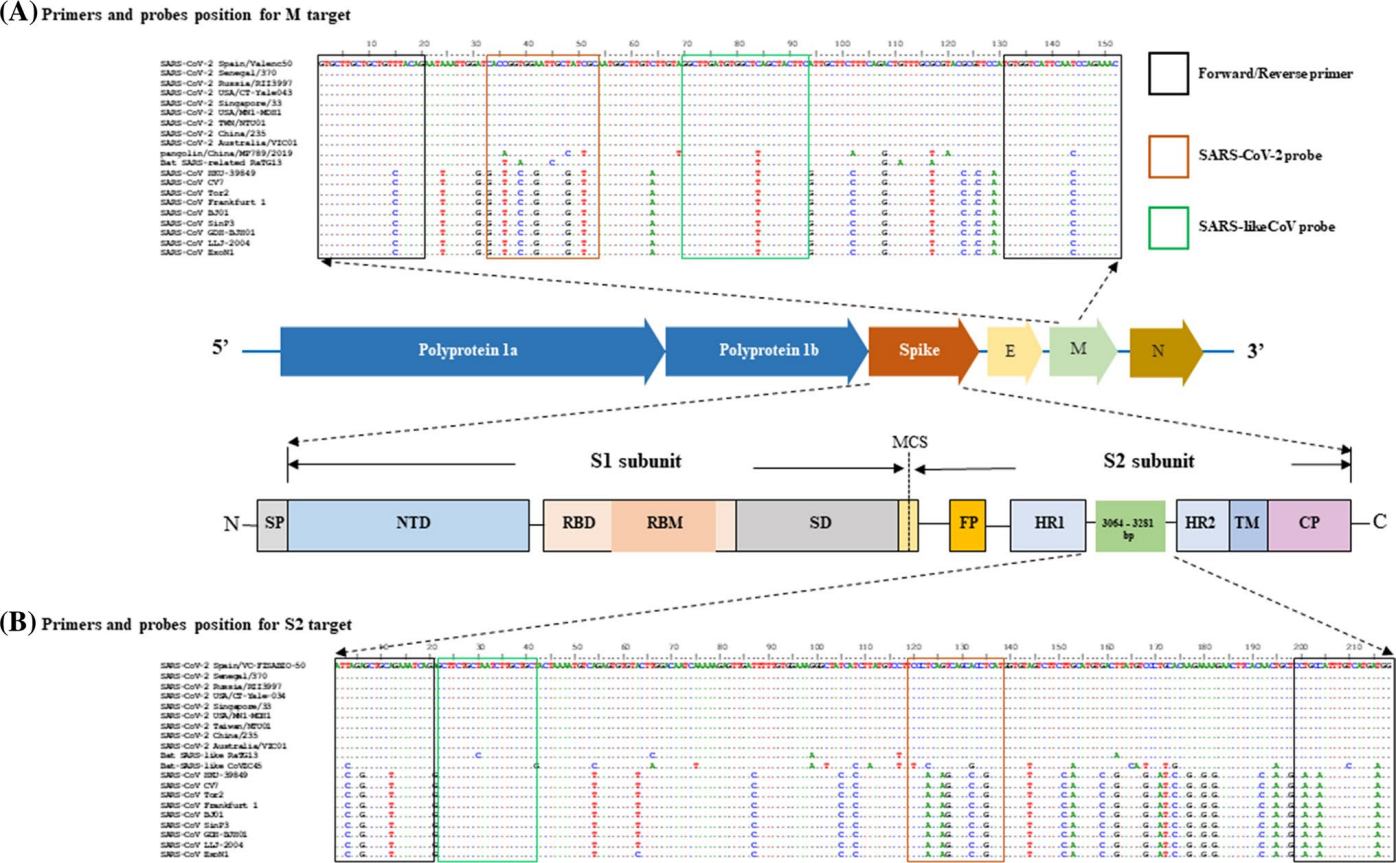
Positions of amplicon targets and oligonucleotide binding regions in the M (**A**) and S (**B**) genes. The panels show oligonucleotide binding regions in rectangular boxes for primer/probe sets A and B (*n* = 20). Dots represent identical nucleotides, and dashes indicate nucleotide deletions compared to the hCoV-19/Spain/Valencia50/2020 sequence, which was the most recently released sequence at the time (GISAID accession number EPI_ISL_420131). The regions encoding domains of the spike protein are annotated. SP, signal peptide; NTD, *N*-terminal domain, *RBD* receptor-binding domain, *RBM* receptor (ACE2)-binding motif, *SD* subdomain, *MCS* multiple cleavage site, *FP* fusion peptide, *HR* heptad repeat, *TM* transmembrane domain, *CP* cytoplasmic domain

After analyzing the alignment of 265 reference sequences, potential target regions on the S and M genes were screened, and sequences satisfying the requirements for our assay design were checked for GC content, melting temperature, potential secondary structure formation, and dimerization using the Oligo Analyzer tool on the Integrated DNA Technology website (https://sg.idtdna.com). The primers and probes designed for the detection of each target were selected and are listed in Table 1. A graphic showing the selected target sequence regions from 20 representative strains (SARS-CoV, SARS-CoV-2, and bat SARS-related CoVs) is presented in Fig. 1. *In silico* analysis was performed to determine the number of mismatches in the primers/probes against SARS-CoV-2 and bat SARS-related-CoVs (Supplementary Table S1).

**Table 1:** Primers and probes for detection of coronaviruses

Primer/probe		Sequence (5'– 3')	Target	Position	Amplicon size (bp)	Reference
Primer/probe set A	SARS-like-CoV-Forward	GTG CTT GCT GCT GTY TAC AG	M gene	26697	152	EPI_ISL_413017
	SARS-CoV-2-Probe	FAM – CAC CGG TGG AAT TGC TAT CGC – BHQ1		26729		
	SARS-like-CoV-Probe	HEX – GAA GTA GCT RAG CCA CAT CAA GCC – BHQ1		– 26789		
	SARS-like-CoV-Reverse	GTT TCT GGR TTG AAT GAC CAC A		– 26848		
Primer/probe set B	SARS-like-CoV-Forward	ATY AGR GCT GCW GAA ATC AG	S gene	24578	218	
	SARS-like-CoV-Probe	Texas red-GCT TCT GCY AAY CTT GCT GC-BHQ2		24599		
	SARS-Cov-2-Probe	Cy5-ATG AGG TGC TGA CTG AGG GA-BHQ2		– 24715		
	SARS-like-CoV-Reverse	CCW TCA TGA CAA ATD GCW GG		– 24795

### Specificity of the multiplex RT-qPCR assay

To determine the specificity of the RT-qPCR assay for distinguishing among SARS-CoV-2, SARS-CoV, and other human-pathogenic CoVs and respiratory viruses, we used 19 viral RNA samples extracted from cultured viruses, including human and swine CoVs, human influenza A (subtype H1N1 and H3N2) and B viruses, dengue virus type 2, parainfluenza virus type 1, human respiratory syncytial virus, and adenovirus type 3. The multiplex RT-qPCR assay showed no cross-reactivity with any of these viruses (Table 2). In particular, SARS-CoVs and bat-associated SARS-like viruses were successfully detected by the SARS-like CoV probe, whereas MERS-CoV and other viruses were not detected by our multiplex RT-qPCR assay. Moreover, we confirmed the performance of the RT-qPCR assay using the viral samples with reference primers and probes targeting the E and RdRp genes, as published in the WHO guidelines [22]. These primer/probe sets could also detect four different SARS-CoV-2 and SARS-CoV isolates as well as SARS-related CoVs from bats (Table 2).

**Table 2:** Analysis of this specificity of the multiplex RT-qPCR assay using cell culture supernatants

Virus	Virus family	Host	Accession number	Mean Ct value (standard deviation)					
				Multiplex assay				WHO reference	
				S gene target		M gene target			
				SARS-CoV-2	SARS- like CoVs	SARS-CoV-2	SARS- like CoVs	RdRp	E
Wild-type SARS-CoV -2 (hCoV-19/South Korea/KUMC01/2020)	*Coronaviridae*	Human	EPI_ISL_413017	15.35 (0.21)	14.03 (0.07)	14.15 (0.13)	14.22 (0.16)	17.31 (0.29)	15.15 (0.45)
Alpha variant SARS-CoV -2 (hCoV-19/South Korea/KDCA0001/2020)	*Coronaviridae*	Human	EPI_ISL_738139	14.23 (0.19)	14.13 (0.12)	13.36 (0.09)	14 (0.11)	17.42 (0.24)	14.08 (0.21)
Beta variant SARS-CoV -2 (hCoV-19/South Korea/KDCA0463/2020)	*Coronaviridae*	Human	EPI_ISL_762992	14.49 (0.09)	14.58 (0.14)	14.37 (0.1)	14.88 (0.15)	18.57 (0.13)	15.24 (0.22)
Delta variant SARS-CoV -2 (hCoV-19/South Korea/KDCA0464/2021)	*Coronaviridae*	Human	EPI_ISL_833249	14.64 (0.18)	15.07 (0.01)	13.96 (0.02)	14.4 (0.02)	18.04 (0.01)	15.18 (0.01)
SARS-CoV (HKU-39849)	*Coronaviridae*	Human	AY278491	N.D	14.75 (0.31)	N.D	14.52 (0.16)	17.2 (0.33)	15.38 (0.26)
Bat SARS-related coronavirus (B18-83)	*Coronaviridae*	Bat	MK991935	N.D	21.92 (0.13)	N.D	22.19 (0.23)	24.88 (0.27)	22.56 (0.17)
Bat SARS-related coronavirus (B18-92)	*Coronaviridae*	Bat	MK991936	N.D	22.18 (0.38)	N.D	22.32 (0.15)	25.05 (0.18)	21.96 (0.35)
Coronavirus NL63 (Korea/CN0601/14)	*Coronaviridae*	Human	MG772808	N.D	N.D	N.D	N.D	N.D	N.D
Coronavirus 229E (Korea/KUMC-9)	*Coronaviridae*	Human	AY386391	N.D	N.D	N.D	N.D	N.D	N.D
Coronavirus OC43 (KBPV-VR-8)	*Coronaviridae*	Human	AY391777.1	N.D	N.D	N.D	N.D	N.D	N.D
MERS-CoV (Korea/KNIH/002_5_2015)	*Coronaviridae*	Human	KT029139	N.D	N.D	N.D	N.D	N.D	N.D
PEDV (Korea/SM98)	*Coronaviridae*	Swine	GU937797	N.D	N.D	N.D	N.D	N.D	N.D
Dengue virus type 2 (Korea/DENV-2/KBPV-VR-29)	*Flaviviridae*	Human	KP406804	N.D	N.D	N.D	N.D	N.D	N.D
Human H3N2 influenza A virus (Korea/37/2012)	*Orthomyxoviridae*	Human	KT889146	N.D	N.D	N.D	N.D	N.D	N.D
Human H1N1 influenza A virus (California/04/09)	*Orthomyxoviridae*	Human	GQ117044	N.D	N.D	N.D	N.D	N.D	N.D
Influenza B virus (B/Brisbane/60/2008)	*Orthomyxoviridae*	Human	FJ766842	N.D	N.D	N.D	N.D	N.D	N.D
Parainfluenza virus 1 (Korea/KUMC-44)	*Paramyxoviridae*	Human	MG255129	N.D	N.D	N.D	N.D	N.D	N.D
Human respiratory syncytial virus (HRSV-A/IC688/12)	*Pneumoviridae*	Human	KP663728	N.D	N.D	N.D	N.D	N.D	N.D
Adenovirus type 3 (KUMC-62)	*Adenoviridae*	Human	KY320276	N.D	N.D	N.D	N.D	N.D	N.D
Control (double-distilled water)				N.D	N.D	N.D	N.D	N.D	N.D

### Detection limit of the multiplex RT-qPCR assay based on *in vitro*-transcribed mRNA and SARS-CoV-2 virions

**Fig. 2:**
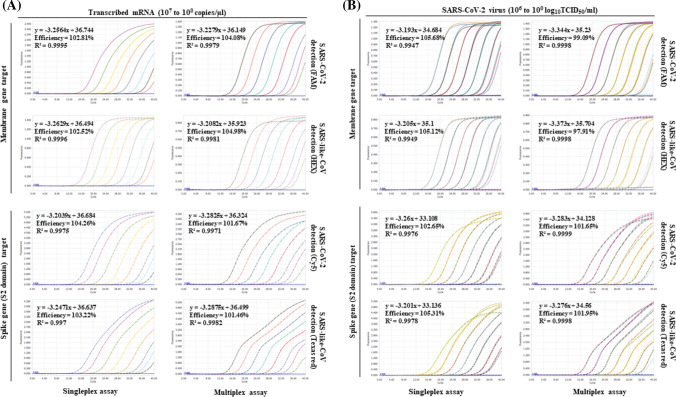
Amplification plots and standard curves of the singleplex and multiplex RT-qPCR assays. The RT-qPCR assay used FAM, Hex, Cy5, and Texas Red dye for the different probes (shown in Table 1). The assays were performed using *in vitro*-transcribed mRNA of wild-type SARS-CoV-2 (KUMC01/2020) and SARS-like CoV. The virus concentration ranged from 5 × 10^7^ to 10^0^ RNA copies per reaction (**A**). The assays were performed using intact viral RNA from SARS-CoV-2, and the range of the serial virus dilution was 6 to 0 log_10_TCID_50_/ml (**B**). The cycle threshold in amplification plots, the correlation coefficient (R2), the slope of the standard curve for the assays, and the efficiency were determined automatically using LightCycler 96 software. Each concentration was tested in triplicate

*In vitro*-transcribed mRNA obtained from the wild-type SARS-CoV-2 (KUMC01/2020) viral RNA template was used for determination of the detection limit of our novel RT-qPCR assay. The detection limit of our multiplex RT-qPCR assay was 5 × 10^0^ mRNA copies per reaction, with Cq values ranging between 35.7 and 36.49, similar to that of the E gene, following the WHO reference protocol (Cq value, 35.86) (Supplementary Table S2). To determine the detection limit of our RT-qPCR assay, a standard curve was made using the Cq values obtained using a tenfold serial dilution of transcribed mRNA from 5 × 10^7^ to 5 × 10^0^ copies/reaction, and the efficiency of the singleplex assay was found to be 102.52–104.26%, compared with 101.67–104.98% for the multiplex assay (Fig. 2A). We confirmed the detection limit of the multiplex RT-qPCR assay using SARS-CoV-2 virions. Four SARS-CoV-2 variants (wild-type, alpha variant, beta variant, and delta variant) were used to extract viral genomic RNA. To compare the detection limit of each singleplex and multiplex assay, both assays were performed in triplicate in three independent experiments. The detection limit of our multiplex RT-qPCR assay was 1 × 10^0^ 50% tissue culture infective dose (TCID_50_) per ml of culture supernatant, with Cq values ranging between 34.15 and 35.32, which is similar to that of the WHO reference protocol (Cq values between 34.41 and 36.42) (Supplementary Table S3). The detection limits for all assays, determined using a standard curve of the Cq values derived from a tenfold serial dilution of viral RNA from the original SARS-CoV-2 strain [27], ranged from 1 × 10^6^ to 10^0^ TCID_50_/mL, and the efficiency of the singleplex assay was 102.65-105.68%, compared with 97.91-101.95% for the multiplex assay (Fig. 2B). These results suggested that the multiplex RT-qPCR assay was capable of being used for screening for a broader range of CoVs while maintaining an analytical sensitivity comparable to that of singleplex assays.

### Multiplex RT-qPCR of human clinical samples

To evaluate the diagnostic performance of the multiplex assay, we tested 29 clinical samples, including nasal swabs and sputum from patients with COVID-19 showing symptoms of respiratory disease, including fever, cough, and/or respiratory failure, obtained from Chungnam National University Hospital (Daejeon, South Korea) and 38 negative specimens from healthy donors. Asymptomatic patients were not included in this study. As shown in Supplementary Figure S4, all specimens from patients with COVID-19 tested positive for SARS-CoV-2 (100% sensitivity), and there was no cross-reactivity with any of the other target viruses in our one-step multiplex RT-qPCR assay. We also tested the same samples using the WHO-recommended protocol, which uses RdRp- and E-gene-specific primer/probe sets. The Cq value obtained in our multiplex assay was comparable to that of the assay targeting the E gene and approximately 2 units lower than the assay targeting the RdRp gene using the WHO-recommended procedure. These results suggested that the sensitivity and specificity of the multiplex RT-qPCR assay for SARS-CoV-2 RNA in swabs and sputum were similar to those of the WHO-recommended assay.

## Discussion

In this study, we developed a novel multiplex molecular diagnostic assay based on RT-qPCR targeting the S and M genes to detect SARS-like CoVs and distinguish SARS-CoV-2 from other CoVs. The S glycoprotein of SARS-CoV-2 has about 76% amino acid sequence identity to that of SARS-CoV and approximately 80% identity to those of bat-derived CoVs [28–30], indicating the utility of S glycoproteins as targets for molecular diagnostics and discrimination between human-pathogenic CoVs and related zoonotic viruses. The M glycoprotein from SARS-CoV-2 is highly conserved and shares approximately 90% sequence identity with those of other members of the subgenus *Sarbecovirus*. The newly designed primer/probe sets targeting S and M genes for the detection of SARS-CoV-2 and SARS-like CoVs were shown to successfully detect the corresponding virus in clinical samples from patients with COVID-19 and several different cultured viruses. WHO recently published a list of several RT-qPCR assays to detect SARS-CoV-2 using RNA transcript standards, including ORF1/nsp14, N, E, and RdRp genes. Our one-step multiplex RT-qPCR assays could have applications in the simultaneous wide screening of SARS-related CoVs and in discrimination of SARS-CoV-2 from other SARS-related CoVs.

In this study, we compared the results obtained using our novel multiplex assay with those obtained using singleplex assays. In particular, the multiplex RT-qPCR assay yielded results that were comparable to those obtained using the assay targeting the E gene and yielded Cq values that were approximately two units lower than those obtained using the assay targeting the RdRp gene for the detection of SARS-CoV-2 in cultured viruses (Table 2) and respiratory tract clinical specimens (Supplementary Table S4). Our multiplex assay could also be performed with a reduced number of primer sets, with four channels for reading signals using a commercial real-time PCR instrument, as compared with conventional multiplex RT-qPCR assays. The small number of tests (67) performed for the detection of SARS-CoV-2 in human clinical samples and the two bat-derived SARS-related CoV samples is a limitation of this study. However, our results showed 100% sensitivity, and the results were comparable obtained targeting those of the RdRp and E genes based on WHO guidelines (Table 2 and Supplementary Table S4). Overall, our one-step multiplex molecular diagnostic assay provides consistent and reliable results.

Bats, which are the natural hosts of many viruses associated with emerging infectious diseases, including SARS-CoVs, could have been the origin of past and ongoing human pandemics, particularly through the involvement of intermediate hosts [31]. Furthermore, expansion of the host range of the virus and the high rate of mutation have contributed to the complex evolutionary dynamics of SARS-CoV-2 [32]. Epidemiological surveillance of SARS-CoVs, an important initial step for the prevention of potential pandemic outbreaks in humans, is necessary. Analysis of the number of mismatches in primers/probes against SARS-CoV-2 and bat SARS-related-CoV (Supplementary Table S1) and specific tests using two bat SARS-like CoV strains (Table 2) suggested that the novel multiplex RT-qPCR assay developed in this study may have applications in detection of SARS-like CoVs in human and bat samples.

In summary, we have developed a novel one-step multiplex RT-qPCR assay that could be used in clinical laboratories to detect human-pathogenic SARS-like CoVs, including SARS-CoV and SARS-CoV-2, and other SARS-like CoVs from bats. This may contribute to the public health response and disease control strategies in countries with limited laboratory capacity.

## Supplementary Information

Below is the link to the electronic supplementary material.

Supplementary file1 (DOCX 27 KB)
