# Supplementary material for: Diagnostic performance and clinical feasibility of a novel one-step RT-qPCR assay for simultaneous detection of multiple severe acute respiratory syndrome coronaviruses
Source: Arch Virol. 2022 Feb 9;167(3):871–9. doi: 10.1007/s00705-022-05383-0 (PMC8885489; doi:10.1007/s00705-022-05383-0)
Supplement: Supplementary file 1 — Supplementary file1 (DOCX 27 KB) [file 705_2022_5383_MOESM1_ESM.docx]

**Supplementary Table S1** Number of mismatches between the primers/probes and SARS-CoV-2 and Bat SARS-related-CoV. Numbers in bold show the sum of the mismatches found in the primers/probes used in RT-qPCR. Numbers in brackets are the number of mismatches in the forward primer (FP), probe (P), and reverse primer (RP), following format FP / P / RP. A total of 265 reference strains, including 200 strains representing 10 clades of SARS-CoV-2 and six variants of concern (sequences released up to 2021.Apr.5), 25 strains of SARS-CoV, and 40 strains of non-human SARS-related-CoV from bats and pangolins were used for analysis.

| Primer/probe | Target | Specificity | Total mismatches | | | |
| --- | --- | --- | --- | --- | --- | --- |
|  |  |  | SARS-CoV-2 | | SARS-related-CoVs | |
| Set A | M gene | Pan-SARS | **0** | [0 / 0 / 0] | **0-2** | [0-1 / 0 / 0-1] |
|  |  | SARS-CoV-2 | **0** | [0 / 0 / 0] | **3-8** | [0-1 / 3-6 / 0-1] |
| Set B | S gene  (S2 domain) | Pan-SARS | **0** | [0 / 0 / 0] | **0-4** | [0-1 / 0-1 / 0-2] |
|  |  | SARS-CoV-2 | **0** | [0 / 0 / 0] | **3-9** | [0-1 / 3-5 / 0-2] |

**Supplementary Table S2** Limits of detection of the multiplex RT-qPCR assays using *in vitro*-transcribed mRNA

| Sensitivity | | Target gene | Limit of detection  (mRNA copies/reaction) | Obtained Cq value |
| --- | --- | --- | --- | --- |
|  |  |  |  | *In vitro*-transcribed wild-type mRNA (KUMC01/2020) |
| One-step multiplex assay | SARS-CoV-2 (FAM) | Membrane | 5 × 10^0^ | 36.14 (0.12) |
|  | SARS-like CoV (HEX) |  | 5 × 10^0^ | 35.7 (0.29) |
|  | SARS-CoV-2 (Cy5) | Spike  (S2 domain) | 5 × 10^0^ | 36.32 (0.21) |
|  | SARS-like CoV (Texas red) |  | 5 × 10^0^ | 36.49 (0.18) |
| WHO reference | SARS-CoV-2 (FAM) | Envelope | 5 × 10^0^ | 35.86 (0.15) |

**Supplementary Table S3** Limits of detection of the multiplex RT-qPCR assays using SARS-CoV-2 virions

| Sensitivity | | Target  gene | Limit of detection (TCID_50_/ml) | Obtained Cq value | | | |
| --- | --- | --- | --- | --- | --- | --- | --- |
|  |  |  |  | Wild-type (KUMC01/2020) | Alpha variant (KDCA0001/2020) | Beta variant (KDCA0463/2020) | Delta variant (KDCA0464/2021) |
| One-step multiplex assay | SARS-CoV-2 (FAM) | Membrane | 1 × 10^0^ | 35.14 (0.32) | 35.1 (0.29) | 35.32 (0.1) | 35.18 (0.45) |
|  | SARS-like CoV (HEX) |  | 1 × 10^0^ | 34.62 (0.35) | 34.83 (0.21) | 34.92 (0.12) | 34.79 (0.31) |
|  | SARS-CoV-2 (Cy5) | Spike  (S2 domain) | 1 × 10^0^ | 34.68 (0.3) | 34.56 (0.22) | 34.82 (0.15) | 34.68 (0.21) |
|  | SARS-like CoV (Texas red) |  | 1 × 10^0^ | 34.42 (0.46) | 34.15 (0.24) | 34.48 (0.3) | 34.35 (0.22) |
| WHO reference | SARS-CoV-2 (FAM) | RdRp | 1 × 10^0^ | 36.1 (0.11) | 36.35 (0.13) | 36.42 (0.36) | 36.29 (0.26) |
|  |  | Envelope | 1 × 10^0^ | 34.42 (0.1) | 34.41 (0.33) | 34.61 (0.13) | 34.48 (0.17) |

**Supplementary table 4** Diagnostics validation of the SARS-CoV-2 RT-qPCR multiplex assay using human clinical samples

| Swab  (Sw#) sample | Our detection system | | | | WHO reference | | Sputum  (Sp#)  sample | Our detection system | | | | WHO reference | |
| --- | --- | --- | --- | --- | --- | --- | --- | --- | --- | --- | --- | --- | --- |
|  | SARS-CoV-2_M  (FAM) | SARS-like _M  (Hex) | SARS-like_S2  (Tex-RD) | SARS-CoV-2_S2  (Cy5) | E | RdRp |  | SARS-CoV-2_M  (FAM) | SARS-like _M  (Hex) | SARS-like _S2  (Tex-RD) | SARS-CoV-2_S2  (Cy5) | E | RdRp |
| Sw#1 | N.D | N.D | N.D | N.D | N.D | N.D | Sp#1 | N.D | N.D | N.D | N.D | N.D | N.D |
| Sw#2 | N.D | N.D | N.D | N.D | N.D | N.D | Sp#2 | N.D | N.D | N.D | N.D | N.D | N.D |
| Sw#3 | N.D | N.D | N.D | N.D | N.D | N.D | Sp#3 | N.D | N.D | N.D | N.D | N.D | N.D |
| Sw#4 | N.D | N.D | N.D | N.D | N.D | N.D | Sp#4 | N.D | N.D | N.D | N.D | N.D | N.D |
| Sw#5 | N.D | N.D | N.D | N.D | N.D | N.D | Sp#5 | N.D | N.D | N.D | N.D | N.D | N.D |
| Sw#6 | N.D | N.D | N.D | N.D | N.D | N.D | Sp#6 | N.D | N.D | N.D | N.D | N.D | N.D |
| Sw#7 | N.D | N.D | N.D | N.D | N.D | N.D | Sp#7 | N.D | N.D | N.D | N.D | N.D | N.D |
| Sw#8 | N.D | N.D | N.D | N.D | N.D | N.D | Sp#8 | N.D | N.D | N.D | N.D | N.D | N.D |
| Sw#9 | N.D | N.D | N.D | N.D | N.D | N.D | Sp#9 | N.D | N.D | N.D | N.D | N.D | N.D |
| Sw#10 | N.D | N.D | N.D | N.D | N.D | N.D | Sp#10 | N.D | N.D | N.D | N.D | N.D | N.D |
| Sw#11 | N.D | N.D | N.D | N.D | N.D | N.D | Sp#11 | N.D | N.D | N.D | N.D | N.D | N.D |
| Sw#12 | N.D | N.D | N.D | N.D | N.D | N.D | Sp#12 | N.D | N.D | N.D | N.D | N.D | N.D |
| Sw#13 | N.D | N.D | N.D | N.D | N.D | N.D | Sp#13 | N.D | N.D | N.D | N.D | N.D | N.D |
| Sw#14 | N.D | N.D | N.D | N.D | N.D | N.D | Sp#14 | N.D | N.D | N.D | N.D | N.D | N.D |
| Sw#15 | N.D | N.D | N.D | N.D | N.D | N.D | Sp#15 | N.D | N.D | N.D | N.D | N.D | N.D |
| Sw#16 | N.D | N.D | N.D | N.D | N.D | N.D | Sp#16 | N.D | N.D | N.D | N.D | N.D | N.D |
| Sw#17 | N.D | N.D | N.D | N.D | N.D | N.D | **Sp#17** | **23.48** | **24.04** | **23.21** | **24.32** | **24.34** | **26.49** |
| Sw#18 | N.D | N.D | N.D | N.D | N.D | N.D | **Sp#18** | **24.73** | **25.38** | **24.41** | **25.71** | **25.52** | **28.92** |
| Sw#19 | N.D | N.D | N.D | N.D | N.D | N.D | **Sp#19** | **23.91** | **24.36** | **23.69** | **24.59** | **25.09** | **27.99** |
| Sw#20 | N.D | N.D | N.D | N.D | N.D | N.D | **Sp#20** | **17.09** | **17.73** | **16.78** | **18.05** | **18.5** | **21.23** |
| Sw#21 | N.D | N.D | N.D | N.D | N.D | N.D | **Sp#21** | **19.69** | **20.29** | **19.4** | **20.59** | **21** | **23.17** |
| Sw#22 | N.D | N.D | N.D | N.D | N.D | N.D | **Sp#22** | **26.14** | **26.62** | **25.9** | **26.87** | **26.75** | **29.27** |
| **Sw#23** | **22.88** | **23.37** | **22.64** | **23.62** | **23.88** | **27.33** | **Sp#23** | **15.5** | **16.16** | **15.17** | **16.5** | **16.52** | **19.63** |
| **Sw#24** | **23.24** | **23.75** | **22.99** | **24.01** | **24.17** | **26.53** | **Sp#24** | **21.61** | **22.13** | **21.36** | **22.39** | **22.72** | **24.8** |
| **Sw#25** | **15.78** | **16.4** | **15.47** | **16.71** | **17.25** | **19.93** | **Sp#25** | **32.21** | **32.95** | **31.84** | **33.32** | **32.32** | **34.71** |
| **Sw#26** | **28.1** | **28.56** | **27.87** | **28.8** | **28.61** | **32.37** | **Sp#26** | **20.16** | **20.78** | **19.85** | **21.09** | **21.18** | **24.86** |
| **Sw#27** | **15.53** | **16.17** | **15.22** | **16.49** | **16.74** | **19.95** | **Sp#27** | **19.39** | **19.97** | **19.1** | **20.26** | **20.61** | **23.51** |
| **Sw#28** | **16.8** | **17.44** | **16.48** | **17.77** | **18.17** | **19.65** | **Sp#28** | **15.98** | **16.73** | **15.61** | **17.11** | **17.08** | **19.78** |
| **Sw#29** | **33.35** | **34.16** | **32.95** | **34.57** | **33.81** | **35.17** | **Sp#29** | **27.14** | **27.68** | **26.87** | **27.96** | **27.96** | **30.28** |
| **Sw#30** | **32.35** | **32.67** | **32.2** | **32.83** | **33.14** | **34.44** | **Sp#30** | **32** | **32.82** | **31.59** | **33.24** | **32.46** | **34.7** |
| **Sw#31** | **22.95** | **23.47** | **22.69** | **23.73** | **23.87** | **26.03** |  |  |  |  |  |  |  |
| **Sw#32** | **16.02** | **16.72** | **15.67** | **17.08** | **16.96** | **19.92** |  |  |  |  |  |  |  |
| **Sw#33** | **29.67** | **29.97** | **29.52** | **30.12** | **29.99** | **34.23** |  |  |  |  |  |  |  |
| **Sw#34** | **25.67** | **26.36** | **25.33** | **26.71** | **24.01** | **27.03** |  |  |  |  |  |  |  |
| **Sw#35** | **23.44** | **23.95** | **23.19** | **24.21** | **24.27** | **26.53** |  |  |  |  |  |  |  |
| **Sw#36** | **25.41** | **26.01** | **25.11** | **26.31** | **24.22** | **26.82** |  |  |  |  |  |  |  |
| **Sw#37** | **31.08** | **32.17** | **30.54** | **32.72** | **32.33** | **34.67** |  |  |  |  |  |  |  |
